# Compromised Teeth Preserve or Extract: A Review of the Literature

**DOI:** 10.3390/jcm11185301

**Published:** 2022-09-08

**Authors:** Valentina Cárcamo-España, Nataly Cuesta Reyes, Paul Flores Saldivar, Eduardo Chimenos-Küstner, Alberto Estrugo Devesa, José López-López

**Affiliations:** 1 Department of Oral Medicine, Faculty of Medicine and Health Sciences (Dentistry), University of Barcelona Dental Hospital, University of Barcelona, 08907 Barcelona, Spain; 2 Department of Odontostomatology and Oral Medicine, Faculty of Medicine and Health Sciences (Dentistry), University of Barcelona Dental Hospital, University of Barcelona, 08907 Barcelona, Spain

**Keywords:** periodontal tissue, prognosis, permanent teeth, periodontal dentistry

## Abstract

Multiple systems and associated factors have been described in the literature to assess the prognosis of teeth with periodontal disease. Nowadays there is a tendency among clinicians to consider implants as the best solution after tooth extraction, in cases of teeth with a questionable prognosis. However, the value of the natural tooth must be considered, as the proprioception of the periodontal ligament is preserved, and it adapts to stress during functional loads. We first review the literature focusing on analyzing the factors that should guide decision-making to maintain or extract a tooth with a compromised periodontium. Then, we propose a schematic diagram of prognostic indicators to reflect the main factors to consider and the survival rate that each one represents when preserving or extracting a tooth.

## 1. Introduction

Oral health care is an essential part of general health and provides people with an increased quality of life [1]. Tooth loss is a serious health problem that affects the functional abilities to chew and speak, psychology, aesthetics, and even social interaction [2]. There is currently no standardized tool to assess the general condition of a tooth and predict whether it is likely to have a long half-life [3].

Prognosis involves “the prediction of the course or outcome of an existing disease, based on empirical information, as well as the ability to recover from the disease” [4]. In dentistry, the predictive probability of dental mortality is based on the stability of the supporting tissues [5]. Various authors postulate that the prognosis is complex, established before treatment, and is supported by clinical and radiographic findings, as well as factors related to the patient, and general factors, such as the systemic condition (diabetes mellitus, smoking habit, motivation, and commitment of the patient) and local factors (factors anatomical, caries, furcation involvement, tooth mobility, periodontal support, pulp involvement, and bone loss) [4]. Prognosis is a dynamic process and should be reassessed, according to the progression of treatment and maintenance of the teeth [3,5].

Understanding the complexity of the prognosis in treatment planning would benefit both the patient and the professional when dealing with other patients facing the same clinical scenario. The development of uniform concepts will facilitate dental education and improve patient care [6].

In recent decades, scientific documentation has positioned implants as the first treatment option in edentulous patients, influencing the decision to extract periodontally compromised teeth [7,8,9,10]. In ref. [11], the authors also suggest that proactive or strategic extraction will prevent future bone destruction in a potential area for subsequent implant placement [11]. However, current evidence cannot always support decision-making, especially considering that any extracted tooth will result in alveolar bone resorption, which can occur despite the use of alveolar ridge preservation techniques or immediate implant placement [12,13,14].

On the other hand, the goal of periodontal therapy is the long-term retention of the natural tooth in a healthy, functional, aesthetically acceptable, and painless state [15]. By way of comparison, when an organ is compromised, measures are taken to prevent further damage or reverse it; however, when it involves a tooth, it is the patients and even some professionals who do not seem to value its preservation [16]. The option of retaining natural teeth, and adopting innovative and cost-effective restorative measures, can provide a practical, pragmatic, and predictable solution over time [17].

The comparison between the preservation of the natural tooth and the placement of an implant is difficult since implants should be considered as a treatment for tooth loss and not as a substitute for the tooth [12]. Clinicians are faced with the dilemma of whether to keep and treat a tooth or extract and replace it with a removable or partially fixed prosthesis. They are the ones who establish the prognosis and carry out the corresponding treatment under their criteria [6].

Based on the foregoing, it may be of interest to have a pattern of action against a tooth of doubtful prognosis; for this, it is important to decide between extracting or not extracting, so the objective of this review was to assess what factors should guide decision-making to maintain or extract a tooth with periodontal involvement with questionable prognosis, and to apply this criterion in a schematic diagram proposed by us.

## 2. Materials and Methods

An electronic search of the PubMed/MEDLINE database, Cochrane Library, and EBSCOhost (Medline, Cinahl) was performed, using the following search strategy: ((“periodontitis” [MeSH Terms]) AND (“prognosis” [MeSH Terms])) AND (“tooth” [MeSH Terms]), without the restriction of years, to compare the available evidence about the tooth with periodontal involvement and make treatment decisions based on its prognosis. A manual search for missing articles that might not have been found in the electronic and gray literature was performed on the references of the selected articles.

This review is carried out based on the PRISMA criteria, fulfilling 21 criteria [18]. The research question was formulated according to the following PICOS criteria: Patients = people with periodontal compromised teeth and questionable prognosis, Intervention = extract the teeth, Control = maintain the teeth, Outcome = prognosis factors and evaluate the evolution of the periodontally compromised teeth, and Study design = literature review.

Articles related to teeth with periodontal involvement and unfavorable or poor prognosis and clinical studies (observational, descriptive, clinical case reports) in English or Spanish were included in the review. In contrast, animal studies, in-vitro studies, and literature reviews were excluded. The data of the included studies (when available) were collected by three independent authors (V.C.-E., N.C.R. and P.F.S.): authors, year, place where the study was carried out, number of subjects, mean age with standard deviation, sex, design of the study, type of periodontitis [19] (aggressive: ≤35 years during the first test of the establishment of the disease with attachment loss ≥5 mm and bone loss ≥50% in more than 2 sites; chronic: ≥40 years during the first test of the establishment of the disease [moderate: 3–4 mm attachment loss and 30–50% bone loss; severe: >5 mm probing depth, >50% bone loss and grade 2 and 3 mobility]), number of teeth with periodontitis, rate of survival or prognosis, factors associated with treatment decision-making, and follow-up (in months). Finally, the information was validated by J.L.-L.

The articles were analyzed for risk of bias using the Newcastle Ottawa Scale (NOS), for the evaluation of cross-sectional studies.

## 3. Results

**Figure 1:**
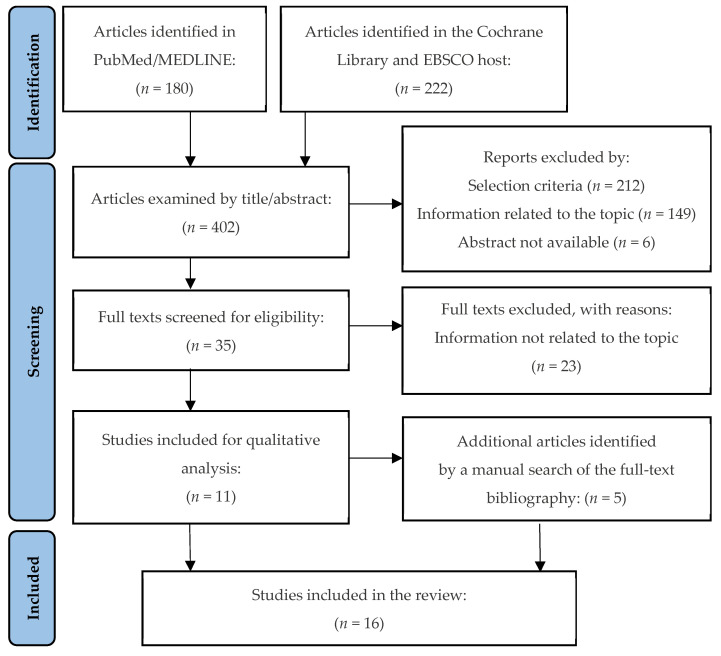
Flowchart showing the synthesis of the bibliographic search, according to the PRISMA guidelines.

The review was carried out from December 2021 to February 2022, both months included. The electronic search in PubMed/MEDLINE, Cochrane Library, EBSCOhost, and a manual search in the bibliography of the selected articles provided 16 articles that met the inclusion criteria [19,20,21,22,23,24,25,26,27,28,29,30,31,32,33,34] (Figure 1) (Table 1 and Table 2). Most of the articles were observational cross-sectional studies [18,19,20,21,22,23,24,28,29,30] and seven were clinical case reports [25,26,27,31,32,33,34]. The main inclusion criteria of the studies reviewed were that patients diagnosed with periodontitis present records from the initial examination, in addition to an accurate periodontal record of the initial condition, immediately after treatment and annually during the maintenance phase. They evaluated the long-term survival of periodontally compromised teeth and associated factors, in patients treated and in periodontal maintenance, including changes in probing depth (mild: 1–3 mm, moderate: 4–6 mm, and severe ≥7 mm), bleeding (mild: <11%, moderate: 11–15% and severe: >15%), and bacterial plaque index (mild: <1, moderate: 1–1.5 and severe: >1.5).

A total of 1.445 patients were examined (Table 3), with an age range of 22–88 years. There was a total of 868 women (60.06%) and 577 men (39.93%). Not all studies evaluated the prognosis of teeth with periodontal involvement (31.25%); however, 14 articles (87.5%) mentioned the reasons for deciding whether to extract or preserve it. Of a total of 26.553 teeth with periodontal involvement, 2.597 were extracted, with the periodontal cause being the most common reason (1.610 teeth [61.99%]), followed by prosthetic reasons (455 teeth [17.52%]) such as caries or crown/root fracture, endodontic complications (86 teeth [3.31%]), and due to unknown or unidentifiable causes by the patient (446 teeth [17.17%]). Thus, 23.956 teeth were preserved, including 144 initially scheduled for extraction. Of these 144 teeth that were preserved, 87 (60.41%) of the patients played a main role in changing the prognosis and making decisions in the final treatment, followed by 57 teeth (39.58%) where the reason was unknown.

In relation to the type of periodontal disease, chronic periodontitis was the most common diseases (nine articles [22,23,25,28,29,30,31,32,33]), followed by aggressive periodontitis (seven articles [19,22,23,24,26,27,29]. Only two of the articles [20,21] mentioned that the patients had severe periodontitis. Finally, of the 16 articles selected, only 5 [22,23,25,27,28] mentioned establishing a prognosis before determining treatment, and the longest follow-up time was 242.4 ± 28.8 months [29].

Among the factors considered prior to making the decision to retain or extract a tooth and subsequent treatment planning, the most common was probing depth ≥5 mm (16 articles [18,19,20,21,22,23,24,25,26,27,28,29,30,31,32,33]), followed by the bacterial plaque index (13 articles [19,20,22,23,25,26,27,29,30,31,32,33,34]), bleeding on probing (9 articles [19,20,21,22,26,27,30,31,34]), smoking >5 years and consumption of ≥10 cigarettes/day (9 articles [19,20,22,23,24,25,26,29,30], grade 2 and 3 tooth mobility (9 articles [20,24,25,27,29,31,32,33,34], and class II and III furcation involvement (8 articles [19,20,21,24,25,27,29,33].

We analyzed the nine cross-sectional observational studies with the Newcastle Ottawa Scale (NOS) (Table 4) and observed that one study had a high risk of bias (50%), three studies had a moderate risk of bias (25%), and five studies had a low risk of bias (0.0–12.5%). In the seven clinical case reports, an assessment of the quality of the evidence was not applied, since blinding of participants and personnel (performance bias) and blinding of outcome assessment (detection bias) were not applicable, associated with incomplete outcome data (attrition bias) and selective reporting (reporting bias).

## 4. Discussion

The decision to keep or extract a periodontally compromised tooth with a hopeless or questionable prognosis is not always easy to predict. Assigning a long-term prognosis is critical, particularly in the dilemma of performing appropriate rehabilitative treatments after periodontal therapy, especially if it involves major prosthetic rehabilitation or implant placement [35]. Lundgren, D. et al. postulate that postponing the insertion of implants in patients susceptible to periodontitis should be considered strategically, optimizing the longevity of the natural dentitions [36] and facilitating a global solution that can reduce the risks of long-term implant treatment [37]. It has been shown that in teeth with a hopeless prognosis or with an indication for extraction, after periodontal treatment, it is possible to stop the progression of the disease to a certain extent and minimize or even prevent tooth loss [12,20,22,24,30]. We must consider that the population is aging, and patients no longer accept removable dentures; they expect that the dentist’s knowledge and skills will allow them to maintain healthy mouths as they age [38]. That is why the demands of the patient must be taken into consideration, but it is the clinician who establishes the treatment plan, in favor or against preserving the tooth. The patient must be fully and adequately informed to have their consent.

After reviewing the selected articles, the decision to keep or extract a tooth depends on several factors, such as the patient’s expectations, control of diabetes mellitus, socioeconomic level, age, oral hygiene, depth of periodontal probing, tooth mobility, root anomalies, furcation involvement, commitment to periodontal treatment and maintenance programs, extensive caries, smoking habit, among others [39,40]. Samet, N. et al. [3] established that the risk factors are divided into biological (systemic condition associated with the immune system and healing, alteration of salivary flow, special needs limiting oral hygiene, high count of *Streptococcus mutans* and *Lactobacillus*, family history, missing teeth), behavior (poor oral hygiene or compromised diet, cariogenic diet, low exposure to fluoride, parafunctional habits, commitment and willingness to adhere to a long-term maintenance protocol, smoking habit), and financial/personal (motivation during treatment, economic resources, time availability, attitude to tooth loss, knowledge about its condition and necessary treatments, aesthetic expectations). For example, in the study by Saminsky, M., et al. [30], the main reason when deciding whether or not the tooth should be extracted was periodontal causes; 11.7% of teeth with periodontal pockets of 4–6 mm and 37.7% with ≥7 mm were extracted (*p* < 0.001). Most patients (32 of 50) received two or more periodontal support treatments per year and multi-rooted teeth (17.9%) showed a higher risk of being extracted compared to single-rooted teeth (3.6%; *p* < 0.001). Among the patient characteristics, it was observed that age is strongly related to tooth loss, especially in patients ≥60 years old (13.9% present risk of extraction; *p* < 0.001). Goh, V., et al. [22] found similar results: sites with probing depth ≥6 mm were positively associated with tooth loss (*p* < 0.002), presenting a greater association when treatment was interrupted for several years (*p* < 0.001).

In this review, several articles postulate various treatment options. However, there are no randomized clinical trials available in the dental literature comparing fixed prostheses in teeth with questionable prognoses with fixed prostheses on implants. In addition, an exact comparison is not possible since each tooth is unique and determined by particular factors. For example, in the study by Tözüm, T.F. et al. [32], after performing the endodontic and periodontal treatment of the compromised tooth, the pain subsided, but the mobility persisted (grade 3). Subsequently, the extraction and intentional reimplantation were carried out, applying an autologous platelet gel inside the alveolus. This allowed a significant gain in clinical attachment level and alveolar bone level, and a total reduction in tooth mobility was observed after 18 months, without observing ankylosis or root resorption.

Another factor previously mentioned is that periodontal support therapy is considered to play an important role in tooth preservation, but the cost and efforts involved are rarely considered [41]. Progression of periodontal disease and reinfection of sites, as well as tooth loss, are possible, especially in patients susceptible to periodontitis [12]. Several factors can affect periodontal healing, such as the presence of morphological defects (a three-walled intraosseous defect will heal better than a one- or two-walled defect), tooth mobility, tissue graft treatments, dentist skills, and level of commitment of the patient [38]. In the study by Graetz, C. et al. [24], after periodontal therapy, the initial mean probing depth was 5.8 ± 2.1 mm and decreased to 3.5 ± 1.1 mm; patients who received adjuvant antibiotic therapy due to persistent inflammation showed an initially greater probing depth of 6.35 ± 2.42 mm and bone loss of >70% in 12.5% (70 teeth).

The fate of a tooth is usually influenced by the treatment planning that involves the entire dentition and the patient’s preferences, with the decision to extract or maintain it largely depending on the dentist, based on their experience and clinical judgment [39,42,43]. To achieve the ideal treatment, there are several factors to be considered during the treatment planning process. These factors include the main demand of the patient; an adequate analysis of the cost-benefit; and risks associated with oral hygiene, tobacco history, and periodontal disease [44]. Su, H. et al. consider that the factor that seems to have the greatest impact on treatment planning is the level of training of the dentist [6]. Clinicians with more than 15 years of experience prefer to perform extractions more frequently than clinicians with less than 5 years of experience [45]. On the other hand, Baba, N.Z. et al. postulate that the treatment decision should be based on satisfying the patient`s wishes and on the importance of evaluating each tooth individually to obtain the treatment with the best result in terms of aesthetics, comfort, function, and cost-effectiveness [46]. In the study by Zafiropoulos, G.G.K., et al. [33], no tooth was extracted in one of the treated patients, since he refused any extraction, opting for 6-monthly maintenance. During the last 4 years of follow-up, the multirooted teeth lost an average of 7.3 mm of clinical attachment, while in the rest of the teeth the loss was only 0.3–0.4 mm. Multirooted teeth with class III furcation involvement had a survival of 8 years.

The placement of implants to replace extracted teeth should be considered acceptable in the case of non-restorable teeth or patients with recurrent periodontal disease, with recurrences after periodontal treatment [46]. Only when the periodontal condition is stabilized and adequate bacterial plaque control is obtained, can the placement of implants be planned as an integral part of the rehabilitation [38]. This should be based on two levels of risk: (1) patient-level: gingival bleeding, the prevalence of residual pockets ≥5 mm, number of missing teeth, loss of attachment/support of the bone level concerning the patient’s age, systemic and genetic condition [46,47], and environmental factors, such as smoking; (2) site level: the presence of residual periapical lesions, alveolar bone height and quality, gingival biotype, the proximity of the anatomical structure, and condition of neighboring teeth (residual periodontal pockets, gingival bleeding and suppuration, tooth anatomy and position, compromise of furca, presence of iatrogenic factors and tooth mobility) [12,48].

It is necessary to expand research related to periodontal and dental prognosis, establish the dental condition at all times, and develop evidence-based treatment strategies [35]. In some cases, it is necessary to integrate the areas of endodontics, periodontics, and orthodontics, to maintain teeth without changing the long-term prognosis [43,49]. When deciding between keeping or replacing a tooth affected by periodontitis, it is important to consider our ability to understand and treat possible future diseases, such as peri-implantitis [44], in which treatment cannot be guaranteed to be predictable [12]. Therefore, it should be discussed whether or not a tooth with a periodontal disease without major restorative treatments should be extracted, assess the potential for success in periodontal treatment, and seriously question the advisability of replacing the tooth [44].

Another factor to consider is tooth extraction for aesthetic reasons, which will only be considered if the prosthetic restoration can significantly improve the aesthetic result and the satisfaction of the patient’s expectations (a key component in the planning of all treatments) [12]. Retaining a tooth may be advantageous in the presence of a thin biotype, unfavorable interproximal bone, or in the presence of a long-standing adjacent implant. It is likely that, after extraction of the tooth with periodontal compromise, the interdental papilla is not present, especially when the distance between the interproximal bone and the proximal contact is greater than 5 mm (>4 mm in thin biotype and >5 mm in thick biotype) [46]. The type of tooth and its position must also be considered; in particular, the molars show less improvement, associated with the complexity of the root anatomy. Martinez-Canut, P. [29] determined that the type of tooth is significantly associated with the risk of tooth loss due to periodontal disease (*p* < 0.001). The risk was multiplied by two in maxillary canines, maxillary incisors, and mandibular lateral incisors; and by seven in maxillary premolars, mandibular central incisors, mandibular canines, and mandibular premolars. In addition, the mandibular first molar was 2.5 times less likely to be lost than the rest of the molars. On the other hand, the absence of adjacent teeth contributed to a better result in teeth with periodontal compromise, since it facilitated the control of bacterial plaque, which must be considered clinically when deciding to extract or maintain a tooth under these conditions [50,51].

**Figure 2:**
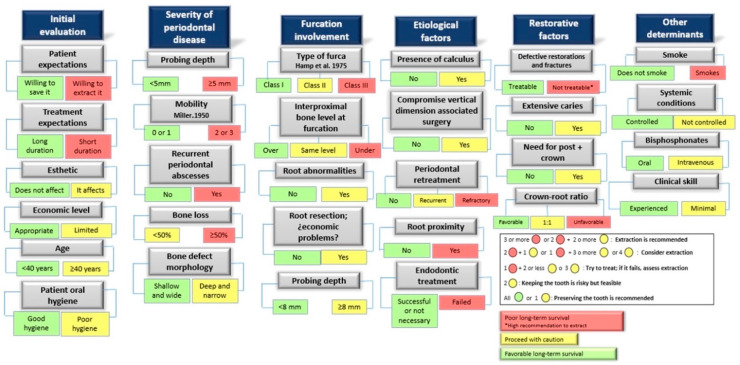
Schematic diagram of the main factors that should guide decision-making to maintain or extract a tooth from a periodontal point of view. Based, with modifications made by the authors, on the schemes initially proposed by Avila, G. et al., 2009 [39]; Nunn, M.E. et al., 2012 [35].

The evidence reflects that the decision to keep or extract a tooth must be multifactorial since it is an irreversible process. The periodontal status and the restorability of the affected tooth should be highlighted as the main factors for prognosis. Taking as reference the publications of Avila, G. et al., 2009 [39] and Nunn, M.E. et al., 2012 [35], we propose a schematic diagram of the prognostic indicators, which reflects the factors to be considered and the survival rate that each one represents, when deciding to keep or extract a tooth (Figure 2).

## 5. Conclusions

In short, and by way of summary, the factors that should guide decision-making to maintain or extract a periodontally compromised tooth include both general patient factors and individual factors of dentition. General factors include biological risk factors, behavioral risk factors, and personal/financial risk factors. Among the individual factors of dentition, we can distinguish periodontal, aesthetic, restorative/endodontic, and prosthetic factors.

## Figures and Tables

**Table 1:** Summary of observational studies evaluated.

Author/Year/Country	Design/Follow-Up (Months)	No. Patients/Gender/Age (Years)	No. Teeth/Type of Tooth/Type of Periodontitis/Prognosis	Factors Associated with Decision-Making	Reason for Extracting/ Preserving
Saminsky, M. et al. (2015) Israel [30]	Cross-sectional 152 ± 25	50 31F/19M 46.6 ± 10.6	1301 M-Pm-C-I ChP NS	Probing depth General health status Smoking habit Periodontal diagnosis Bacterial plaque index Bleeding on probing	151 extracted (96 periodontal causes/55 extensive caries or root fracture)
Goh, V. et al. (2017) China [22]	Cross-sectional 100.4 ± 44.4	65 34F/31M 43.8 ± 11.9	1597 M-Pm-C-I AgP-ChP Good (pdep ≤4 mm)-Fair (pdep ≥5 mm)-Questionable (pdep 6–8 mm), Hopeless (pdep ≥8 mm)-Undetermined	General health status Oral hygiene Use of removable prosthesis Smoking habit Dental visit history Number of teeth Bacterial plaque index Bleeding on probing Probing depth Bone crest level	229 extracted (191 periodontal reasons/23 caries/15 not identifiable by the patient)
De Beule, F. et al. (2017) Belgium [21]	Cross-sectional 197	402 201F/201M 34–88	2559 M* SevP NS	Medical condition Probing Depth Furca engagement Bone loss Gum health Bleeding on probing Tooth type Tooth location	511 extracted (377 periodontal reasons/60 endodontic problems or endo-periodontal lesions/17 fracture/25 caries/1 prosthetic strategy/31 unknown reason)
D’Aiuto, F. et al. (2005) England [20]	Cross-sectional 6	94 50F/44M 46 ± 9	2589 M-Pm-C-I SevP NS	Probing depth Gingival recessions Bacterial plaque index Bleeding on probing Clinical insertion level Furca engagement Tooth mobility Tooth type Smoking habit Periodontal diagnosis	NS
Graetz, C. et al. (2017) Germany [24]	Cross-sectional 208.8 ± 57.6	57 35F/22M 34.7 ± 8	1505 M-Pm-C-I AgP NS	Probing depth Tooth mobility Radiographic bone loss Furca engagement Smoking habit Tooth type Preoperative antibiotic therapy	232 extracted (prosthetic and periodontal reasons)
Machtei, E. & Hirsch, I. (2007) Israel [28]	Cross-sectional 156	93 59F/34M 45.54 ± 1.13	110 (74 multirooted/36 single root) ChP Hopeless	Probing depth Radiographic bone loss	53 extracted 57 saved (the decision was made by the patient without influence from the dentist)
Bäumer, A. et al. (2011) Germany [19]	Cross-sectional 126	84 68F/16M 30.8 ± 4.1	2154 M*-Pm-C-I AgP NS	Smoking habit History of periodontal disease Dental status Probing depth ≥5 mm Clinical insertion level Bleeding on probing Suppuration on probing Furca engagement Gingival index Bacterial plaque index Educational level Dental care compliance	166 extracted (unknown reason)
Graetz, C. et al. (2011) Germany [23]	Cross-sectional 193 ± 54	68 (34AgP/34ChP) 28F (11AgP/17ChP) 40M (23AgP/17ChP) 33.3 ± 4.1AgP 51.6 ± 7.4ChP	923AgP/874ChP M-Pm-C-I AgP-ChP Good (bone loss <50%)-Questionable (bone loss ≥50%- < 70%)-Hopeless (bone loss ≥70%)	Smoking habit Radiographic bone loss ≥50% Probing depth Bacterial plaque index Preoperative antibiotic therapy	142AgP extracted 133ChP extracted (112AgP-48ChP periodontal reasons/the rest due to endodontic involvement, caries, prosthetics, fracture, or unknown reason)
Martinez-Canut, P. (2015) Spain [29]	Cross-sectional 242.4 ± 28.8	500 344F/156M 22–74	12.830 M*-Pm-C-I AgP-ChP NS	Health condition Smoking habit Bacterial plaque index Probing depth >6 mm Gum recession Furca engagement Tooth mobility 2–3 Radiographic bone loss >50% Root crown ratio 1/1 Root anatomy Periodontal diagnosis	875 extracted (515 periodontal disease/172 non-restorable caries/75 root and/or coronary fracture/26 endodontic complications/85 strategic extraction for prosthetic and orthodontic considerations)

F: female, M: male, AgP: aggressive periodontitis, ChP: chronic periodontitis, SevP: severe periodontitis, EndP: endoperiodontal injury, M: molar tooth, M*: except third molar, Pm: premolar tooth, C: canine tooth, I: incisive tooth, MxLI: maxillary lateral incisor, MdCI: mandibular central incisor, pdep: probing depth, NS: not specified.

**Table 2:** Summary of clinical case reports evaluated.

Author/Year/Country	Design/Follow-Up (Months)	No. Patients/Gender/Age (Years)	No. Teeth/Type of Tooth/Type of Periodontitis/Prognosis	Factors Associated with Decision-Making	Reason for Extracting/ Preserving
Grigorie, M.M. et al. (2021) Romania [25]	Case report 48	1 1F 62	27 M-Pm-C-I ChP Hopeless (pdep >8 mm, with class II or higher furcation involvement and bone loss ≥70%).	Smoking habit Probing depth ≥5 mm Bony vertical defects Furca engagement Tooth mobility Bacterial plaque index Tooth migration Reduced periodontal support Infrabony defect	1 extracted (caries and endodontic complications)
Kavarthapu, A. & Malaiappan, S. (2019) India [27]	Case report 9	1 1F 28	1 M AgP Bad	Probing depth >8 mm Bleeding on probing Bacterial plaque index Grade II furcation involvement Vertical bone loss Purulent discharge Tooth mobility grade 2 Negative pulp vitality Apical radiolucency	The tooth was saved (patient compliance)
Seshima, F. et al. (2016) Japan [31]	Case report 14	1 1M 66	27 M-Pm-C-I ChP NS	Probing depth ≥7 mm Bone loss Bleeding on probing Bacterial plaque index Blood glucose levels Tooth mobility	1 extracted (prophylactic reasons: impacted tooth)
Zafiropoulos, G.G.K. et al. (2011) Germany [33]	Case report 180/84	2 1F/1M 33/39	28/26 M-Pm-C-I ChP NS	Probing depth Bleeding on probing Bacterial plaque index Furca engagement Radiographic bone loss ≥50% Tooth mobility Clinical insertion level Periodontal pathogens	Case 1: all were preserved (the patient rejects any extraction); Case 2: 21 extracted (advanced bone loss and/or dental mobility)
Zucchelli, G. (2007) Italy [34]	Case report 12–36	1 1F 39	1 MxLI EndP NS	Probing Depth Bacterial plaque index Bleeding on probing Clinical insertion level Gingival recession ≥3 mm Bone loss Tooth mobility grade 3 Radiographic radiolucency Negative vitality test Absence of fillings	53 extracted (43 multirooted/10 single root); 57 saved (31 multirooted/26 single root) (unknown reason)
Tözüm, T.F. et al. (2006) Turkey [32]	Case report 18	1 1M 42	1 MdCI ChP NS	Bacterial plaque index Clinical attachment level ≥6 mm Tooth mobility grade 3 Negative vitality test Probing depth ≥4 mm Radiographic bone loss Extrusion Keratinized gingiva ≥2 mm	1 saved (upon advice from the dentist to consider a new treatment option)
Kamma, J.J. & Baehni, P.C. (2003) Greece [26]	Case report 60	25 14F/11M 34.3 ± 2.5	NS NS AgP NS	Smoking habit Bacterial plaque index Gingival index Bleeding on probing Suppuration on probing Probing depth Clinical insertion level Radiographic bone loss	29 extracted (18 due to furca involvement/the rest unknown reason)

**Table 3:** Summary of demographic data and teeth evaluated.

Variable		Total
Gender	Women	868 (60.06%)
	Men	577 (39.93%)
Total patients		1.445
Age range		22–88 years
Total teeth examined		26.553
Teeth extracted		2.597
Periodontal reasons		1.610 (61.99%)
Prosthetic reasons		455 (17.52%
Endodontic complications		86 (3.31%)
Unknown or unidentifiable reason		446 (17.17%)
Teeth preserved (no initial commitment)		23.812
Teeth preserved (with initial commitment)		144
The patient made the final decision		87 (60.41%)
Unknown or unidentifiable reason		57 (39.58%)

**Table 4:** The table shows the risk of bias criteria using the adapted Newcastle Ottawa Scale (NOS) for cross-sectional studies. If the criterion is met, a green dot is placed in the box, otherwise, if it is not met, a red dot is placed. Studies with a total score of 7 or 8 green points were considered a low risk of bias; 6 green dots were considered to be at medium risk of bias; 5 green dots or less were judged to be at high risk of bias.

Item	Saminsky, M. et al., 2015 [30]	Goh, V. et al., 2017 [22]	De Beule, F. et al., 2017 [21]	D’Aiuto, F. et al., 2005 [20]	Graetz, C. et al., 2017 [24]	Machtei, E. & Hirsch, I., 2007 [28]	Bäumer, A. et al., 2011 [19]	Graetz, C. et al., 2011 [23]	Martinez-Canut, P., 2015 [29]
Selection									
1. Is the sample representative of the target average population?	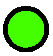	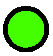	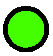	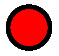	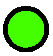	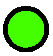	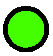	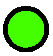	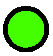
2. Was the sample size justified and satisfactory?	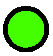	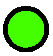	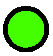	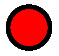	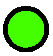	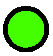	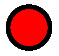	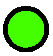	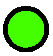
3. It was established which subjects would be included and was the inclusion range satisfactory?	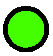	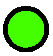	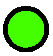	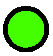	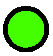	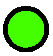	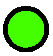	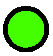	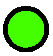
Comparability									
1. Were anthropometric measurements adequately adjusted for age and gender?	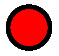	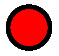	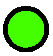	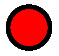	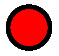	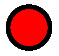	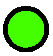	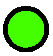	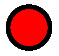
2. Were other factors such as race/ethnicity, educational level, habits, probing depth, survival rate, etc. adequately adjusted?	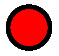	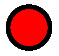	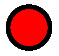	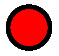	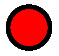	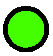	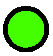	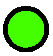	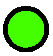
Results									
1. Was the result established independently and with data linkage?	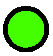	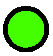	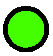	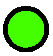	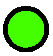	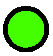	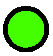	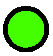	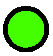
2. Was the result determined by a self-report?	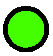	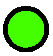	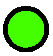	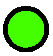	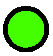	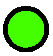	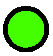	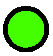	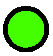
3. The statistical test used to analyze the information is clearly described and appropriate, and the measures of the association presented include confidence intervals and level of probability (*p*-value)?	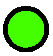	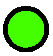	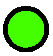	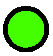	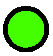	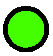	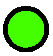	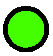	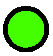
Total	6	6	7	4	6	7	7	8	7

## Data Availability

Not applicable.
